# Induction of Posttranslational Modifications of Mitochondrial Proteins by ATP Contributes to Negative Regulation of Mitochondrial Function

**DOI:** 10.1371/journal.pone.0150454

**Published:** 2016-03-01

**Authors:** Yong Zhang, Zhiyun Zhao, Bilun Ke, Lin Wan, Hui Wang, Jianping Ye

**Affiliations:** 1 Antioxidant and Gene Regulation Laboratory, Pennington Biomedical Research Center, Louisiana State University System, Baton Rouge, LA 70808, United States of America; 2 Laboratory of Transplantation Immunology, Regenerative Medicine Research Center, West China Hospital, Sichuan University, Chengdu, China; 3 Collaborative Innovation Center of Molecular Diagnosis and Laboratory Medicine in Henan Province, School of Laboratory Medicine, Xinxiang Medical University, Xinxiang 453003, China; Warren Alpert Medical School of Brown University, UNITED STATES

## Abstract

It is generally accepted that ATP regulates mitochondrial function through the AMPK signaling pathway. However, the AMPK-independent pathway remains largely unknown. In this study, we investigated ATP surplus in the negative regulation of mitochondrial function with a focus on pyruvate dehydrogenase (PDH) phosphorylation and protein acetylation. PDH phosphorylation was induced by a high fat diet in the liver of obese mice, which was associated with ATP elevation. In 1c1c7 hepatoma cells, the phosphorylation was induced by palmitate treatment through induction of ATP production. The phosphorylation was associated with a reduction in mitochondria oxygen consumption after 4 h treatment. The palmitate effect was blocked by etomoxir, which inhibited ATP production through suppression of fatty acid β-oxidation. The PDH phosphorylation was induced by incubation of mitochondrial lysate with ATP in vitro without altering the expression of PDH kinase 2 (PDK2) and 4 (PDK4). In addition, acetylation of multiple mitochondrial proteins was induced by ATP in the same conditions. Acetyl-CoA exhibited a similar activity to ATP in induction of the phosphorylation and acetylation. These data suggest that ATP elevation may inhibit mitochondrial function through induction of the phosphorylation and acetylation of mitochondrial proteins. The results suggest an AMPK-independent mechanism for ATP regulation of mitochondrial function.

## Introduction

Mitochondria utilize glucose and free fatty acid (FFA) in the production of ATP. As a product of mitochondria, ATP may inhibit mitochondrial function in the feedback regulation. This ATP activity may play a role in the mechanism of insulin resistance under over-supply of free fatty acids (FFAs). Specifically, ATP may mediate FFA signal to inhibit glucose utilization in mitochondria to cut down glucose demand in cells in the mechanism of insulin resistance as suggested by Randle, et al. years ago [1]. In the mechanism, ATP may use two pathways in the feedback regulation of mitochondria. The first is down-regulation of the AMPK (AMP-activated kinase) pathway, in which ATP surplus leads to inhibition of AMPK activity for suppression of mitochondrial function [2, 3]. AMPK is known to promote mitochondria activity under energy-deficient conditions in cells. The second is inhibition of PDH (pyruvate dehydrogenase, pyruvate dehydrogenase complex, PDC) activity to limit glucose influx into mitochondria. There are two possibilities in the inhibition of PDH by ATP: allosteric modification of PDH [1] and induction of PDH phosphorylation [4]. ATP is able to induce PDH phosphorylation through direct activation of PDK (PDH kinase) enzyme in vitro [4]. However, the role of ATP in the induction of PDH phosphorylation remains to be established in the context of obesity.

The PDK-PDH pathway represents an AMPK-independent pathway in the regulation of mitochondrial function [5]. PDH is a mitochondrial protein complex containing three types of enzymes [5]: pyruvate dehydrogenase E1 (PDHe1), dihydrolipoamide transacetylase E2 (PDHe2) and dihydrolipoamide dehydrogenase E3 (PDHe3). PDH stimulates acetyl-CoA production from pyruvate in the glucose catabolism pathway. This activity is required for insulin-induced glucose utilization. Inhibition of PDH activity by phosphorylation of the PDHe1 alpha subunit is a mechanism of insulin resistance in starvation and diabetes [5–8]. The phosphorylation is catalyzed by PDKs at three serine residues (S232, S293, and S300). The phosphorylation is enhanced by acetylation in the subunit [9]. Although PDK expression is a mechanism of PDH phosphorylation, an expression-independent mechanism remains unknown. ATP may induce the phosphorylation without induction of PDK expression. The possibility is supported by evidence that a high level of intracellular ATP is associated with a high risk of insulin resistance in human [10] and mice [11]. To test this possibility, we examined ATP in the induction of PDH phosphorylation. There is no report about interaction of PDK and AMPK in the regulation of mitochondrial function to our knowledge.

Lysine acetylation is an important mechanism in the regulation of enzyme activities in variety of metabolic pathways [12–14]. The activity is documented in mitochondria in several models including SIRT3 knockout mice [15–17]. Acetylation requires both acetyl-CoA (substrate of acetyltransferase) and ATP (energy source). Over production of ATP and acetyl-CoA may promote the acetylation in mitochondria. The role of acetyl-CoA has been investigated in the pathogenesis of hepatic insulin resistance in mouse models in a recent study [18]. However, the study does not provide a mechanism for acetyl-CoA in insulin resistance. Given that acetyl-CoA promotes ATP production in mitochondria, we propose that ATP may mediate acetyl-CoA activity in the induction of acetylation. We propose that ATP may contribute to insulin resistance through modification of mitochondrial proteins by phosphorylation and acetylation. Inhibition of ATP production is a promising approach in the treatment of insulin resistance as suggested by the activities of anti-diabetic medicines [19–21].

We hypothesize that ATP may inhibit mitochondria function through direct induction of phosphorylation as well as acetylation of mitochondrial proteins. This possibility was investigated in the liver of diet-induced obese (DIO) mice and 1c1c7 hepatoma cells in this study.

## Materials and Methods

### Animal

Male C57BL/6 mice were purchased from the Jackson Laboratory (Bar Harbor, ME) at 6 wks of age. The mice were maintained in the animal facility of the Pennington Biomedical Research Center with a 12:12-h light-dark cycle, constant room temperature (22–24°C), free access to water and diet. Mice were fed a high fat diet (HFD, D12331, 36% w/w or 58% calories in fat, Research Diets, New Brunswick, NJ) at 8 wks (C57BL/6J) to generate a diet-induced obese (DIO) model as described elsewhere [22]. The control mice were fed a Chow diet (5001, containing 5% w/w or 11% calories in fat, Labdiet, St. Louis, MO). All procedures were performed in accordance with the National Institutes of Health guidelines for the care and use of animals and were approved by the Institutional Animal Care and Use Committee (IACUC) at the Pennington Biomedical Research Center. The mouse livers were collected at 12 wks on HFD for analysis of mitochondrial proteins.

### Hepatic steatosis

Hepatic steatosis was determined by liver size, lipid content of hepatocytes in histology and triglyceride content per gram liver tissue. Hematoxylin and eosin (H&E) staining of liver tissue and triglyceride assay was performed using protocols as described elsewhere [23].

### Cells and reagents

Cell line Hepa-1c1c7 (CRL-2026^™^) was purchased from the American Type Culture Collection (Manassas, VA). The cells were maintained in DMEM supplemented with 10% fetal calf serum. Sodium palmitate (P9767), etomoxir (E1095), calyculin (C5552), acetyl-CoA (A2056) and ATP (A6559) were purchased from Sigma (St. Louis, MO). Antibodies to phosphorylated PDH e1 alpha subunit (S293) (ab177461), PDHe1 alpha subunit (ab110330), PDK2 (ab68164), PDK4 (ab89295), and tubulin (ab7291) and anti-voltage dependent anion channel (VDAC1, ab14734) were obtained from Abcam (Cambridge, MA). Antibody to acetylated lysine (#9814) was from Cell Signaling (Danvers, MA).

### Palmitate, ATP and acetyl-CoA treatment

1c1c7 cells were passed every two days in mycoplasma free medium. The cells were starved overnight at 90% confluence in the 10 cm plate in DMEM containing 0.25% BSA and then treated with BSA-conjugated sodium palmitate at the final concentration of 300 μM. BSA was used in the control for palmitate. Etomoxir (50 μM) was used to inhibit β-oxidation with 4 h treatment. Calyculin (20 nM) was used to induce phosphorylation in cells with 1 h treatment. Mitochondrial lysate was made from freshly-isolated mitochondria and kept in -70°C until experiment. The lysate was incubated with ATP (1–10 μM/g) or acetyl-CoA (1–500 μM) for 30 min at 37°C to induce protein phosphorylation and acetylation.

### Mitochondria isolation

Mitochondria were isolated from live cells or fresh liver tissue using the standard protocol [24]. Mitochondrial isolation buffer is composed of 70 mM sucrose, 210 mM mannitol, 5 mM HEPES, 1 mM EGTA and 0.5% fatty acid-free BSA (pH 7.2). Mitochondrial assay solution (MAS) comprises 70 mM sucrose, 220 mM mannitol, 10 mM KH_2_PO_4_, 5 mM MgCl_2_, 2 mM HEPES, 1 mM EGTA and 0.2% fatty acid-free BSA, pH 7.2 at 37°C. A stock solution (2X) of MAS was prepared for dilution of substrates, ADP and respiration reagents. Stocks of succinate and ADP were made in H_2_O and adjusted to pH 7.2 with potassium hydroxide. Stocks of 10 mM FCCP (carbonyl cyanide 4-trifluoromethoxy phenylhydrazone), 2 mM rotenone, 5 mg/ml oligomycin and 40 mM antimycin A were made in DMSO.

Cells were homogenized using a glass-Teflon potter, and centrifuged at 600 *g* for 10 minutes at 4°C. The supernatant was then centrifuged for 10 minutes at 7,000 *g* at 4°C, and the pellet was collected as mitochondria after washing with ice-cold buffer. Mitochondria were suspended and then used in functional analysis. Mitochondria were sonicated in lysis buffer in the preparation of mitochondrial lysate. Protein concentration was determined by the BCA (bicinchoninic acid) method using BSA as a standard.

### Phosphorylation and acetylation

Liver tissues were collected from DIO mice at 12 wks on HFD. Mitochondrial lysate was used at 50 μg protein/sample and resolved on 8% SDS-PAGE gel by electrophoresis. The protein was blotted onto nitrocellulose membrane. Blots were then blocked in 5% nonfat milk buffer containing 0.05% Tween-20, rinsed in PBS (pH 7.4) as described elsewhere [25], The membrane was incubated with the following antibodies: phospho-PDHe1 (1:10000), PDHe1 (1:1000), anti-acetylated lysine (1:1000), and VDAC1 (1:1000). VDAC1 was used as a loading control of mitochondrial protein. The signal was visualized using chemiluminescence. The specific signal was scanned and analyzed for optical densities in quantification using the image-processing and analysis system (Bio-Rad, Hercules, CA).

### Oxygen consumption rate (OCR)

All OCR assays were performed using XF24 Extracellular Flux Analyzer (Seahorse Bioscience, North Billerica, MA). The assay is based on fluorimetric detection of O_2_ and H^+^ levels via a sensor cartridge that is equipped with four reagent injectors. Mitochondrial substrates and compounds were prepared at 10× final concentrations in 1× MAS buffer. The reagents were loaded into the injection ports before mitochondria loading to the XF plate. The loaded cartridge was calibrated before the assay. To minimize variability among the wells, mitochondria were first diluted by 10 times in cold MAS buffer with substrate (succinate), and then diluted again to the final concentration. The mitochondrial suspension (50 μl) was delivered into each well of XF plate (except for background correction wells) on ice. The plate was centrifuged at 2000 *g* for 20 minutes at 4°C, and then loaded with 450 μl/well of substrate in 1× MAS. The mitochondria were observed briefly under a microscope to ensure consistent adherence to the well bottom, then placed at 37°C for 6–8 minutes to allow the plate to warm up. The plate was used in the OCR measurement according to the protocol.

### ATP assay

ATP was measured using the ATP Determination Kit (Life technologies, Eugene, Oregon) immediately after mitochondria isolation. For all experiments, ATP standard curves were run in the range of 1 μM to 1mM and the correlation coefficient was 0.990 or higher. The samples were added to the reaction mixture and the luminescent signal was measured by the luminometer (Bio-tek, Winooski, VT). The ATP concentration was normalized with the mitochondrial protein.

### Acetyl-CoA assay

The acetyl-CoA content was determined immediately after isolation of the mitochondria using the PicoProbe acetyl-CoA assay kit (ab87546, Abcam). Briefly, acetyl-CoA standard curve was made in the range of 0–100 pM and the correlation coefficient was 0.990 or higher. Protein was removed in the sample using the perchloric acid protocol and the supernatant was neutralized with 3 M KHCO_3_. The CoASH Quencher and Quencher remover were added into the sample to correct the background generated by free CoASH and succ-CoA. The sample was then diluted with the reaction mix, and the fluorescence signal was measured at Ex/Em = 535/589 nm with Spectra max Gemini XPS (Molecular Devices, Sunnyvale, CA). The relative acetyl-CoA concentration was normalized with the mitochondrial protein.

### PDH enzyme assay

PDH activity was measured according to the protocol by PDH activity assay kit (ab109902, Abcam). Mitochondria were diluted and added into the microplate. After incubation in the plate for 3 hours at room temperature, the samples were stabilized and incubated with assay buffer. The fluorescence was measured at 450 nm for 30 minutes with 30 seconds interval among each measurement, and the slope of the line indicated the PDH activity.

### Statistical analysis

In this study, the data were presented as the mean ± SEM from multiple samples. In cell culture study and immunoblotting, the experiments were repeated at least three times. The representative immunoblot blot is presented. In the statistical analysis, two-tailed, unpaired Student’s t test was used in analysis of in vitro data, and one-way ANOVA was used in analysis of in vivo data with significance P <0.05.

## Results

### ATP and PDH phosphorylation levels in liver of DIO mice

**Fig 1:**
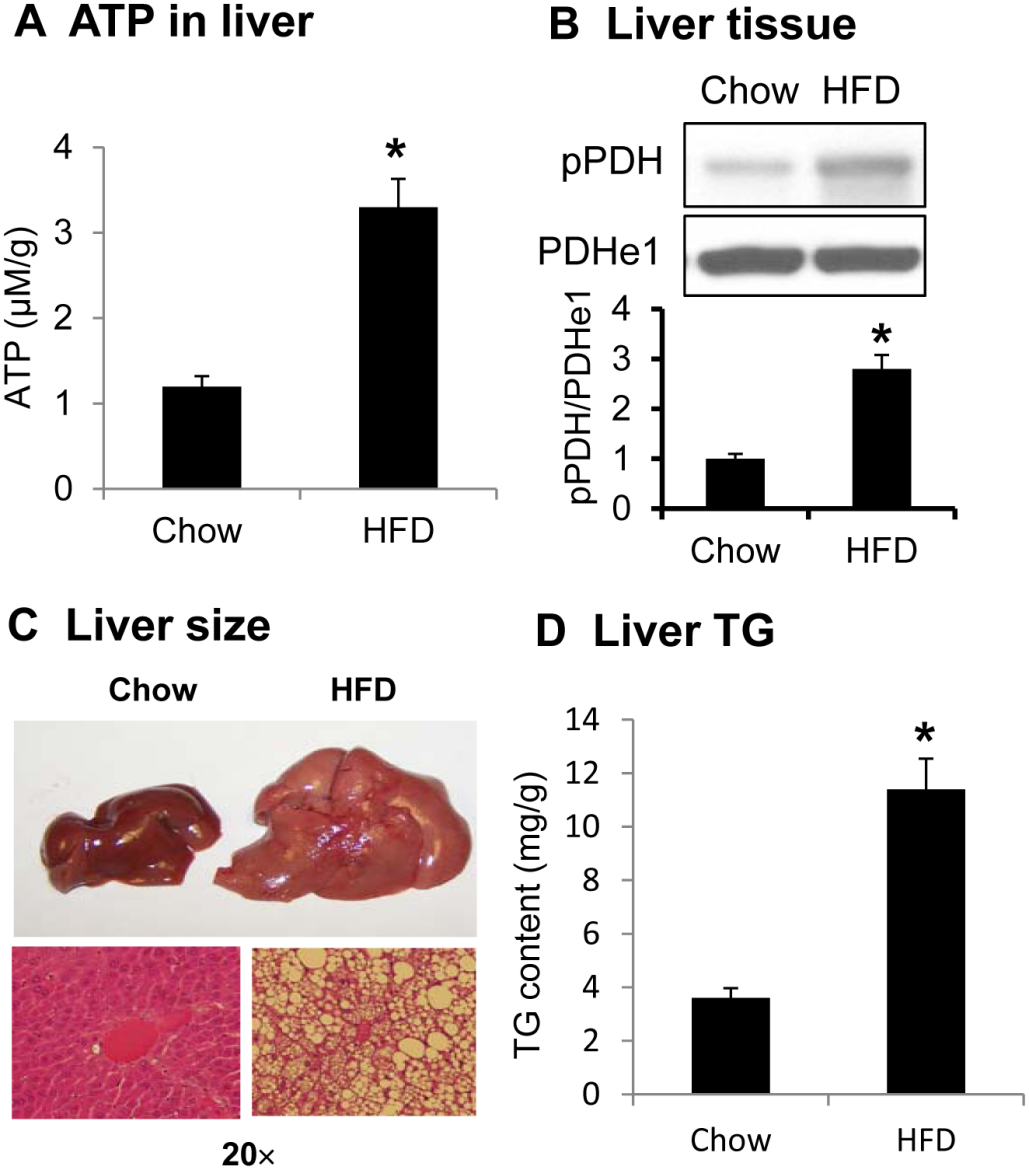
ATP and PDH phosphorylation in liver of DIO mice. A. ATP in liver tissue. ATP was determined in fresh liver tissue of mice on Chow and HFD. Liver was collected from mice after overnight fasting at 12 wks on HFD. B. PDH phosphorylation in liver. PDH phosphorylation was examined in the liver tissue lysate in Western blotting. C. Liver size and histology. D. Triglyceride (TG) content in liver. In the bar figure, the result represents mean ± SE (n = 5). * p<0.05 compared with the control.

Diet-induced obese mouse is a well-established model in the study of insulin resistance. We use the model routinely in our laboratory in the study of type 2 diabetes. ATP was tested in the liver of DIO mice at 12 weeks on HFD. In the fresh liver tissue, ATP was increased by 2 folds (Fig 1A). The increase was associated with enhanced phosphorylation of PDH (Fig 1B). The phosphorylation was determined in E1 subunit of PDH by Western blotting. Hepatic steatosis was observed in DIO mice as indicated by the liver size, histology and triglyceride content (Fig 1C and 1D). The data suggests that intracellular ATP is positively associated with PDH phosphorylation status in liver of obese mice.

### Induction of PDH phosphorylation by ATP

**Fig 2:**
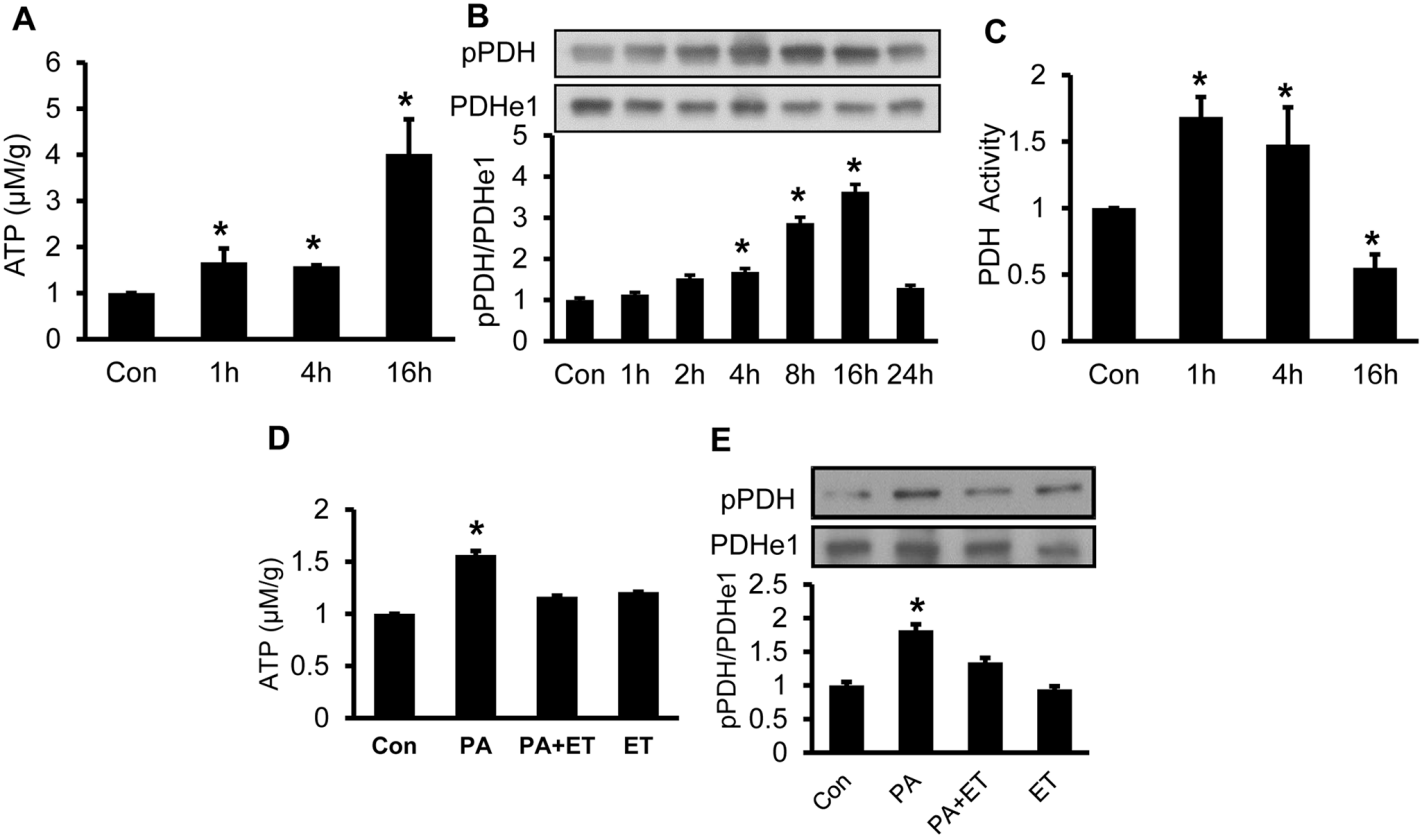
Induction of PDH phosphorylation by ATP in cells. A. Palmitate induction of ATP. ATP was tested in the fresh cell lysate after palmitate (300 μM) treatment at 1, 4 and 16 h. B. PDH phosphorylation. The test was performed at multiple time points with palmitate (300 μM) treatment as indicated. C. PDH enzyme activity. The activity was determined in the cell lysate. D. ATP inhibition by etomoxir (ET). ET (50 μM) was used to block β-oxidation in cells treated with palmitate (300 μM, 4 h). ATP was determined in cell lysate. E. Prevention of PDH phosphorylation by ET. In the bar figure, the result represents mean ± SE (n = 3). * p<0.05 compared with the control (0 point).

To test the possible role of ATP in the induction of PDH phosphorylation, ATP was tested in cells, in which 1c1c7 hepatoma cells were treated with palmitate to induce intracellular ATP. Palmitate was conjugated with BSA and used as a free fatty acid in the study. An increase in ATP was observed in the cells at 1 h of palmitate treatment and the peak was observed at 16 h (Fig 2A). An induction of PDH phosphorylation was observed in the cells, and the change was in a time-dependent manner with a peak at 16 h followed by a decrease at 24 h (Fig 2B). The elevated phosphorylation was associated with an increase in the catalytic activity of PDH at 1–4 h and then decrease at 16 h (Fig 2C). The association of ATP elevation and PDH phosphorylation suggest that ATP may regulate PDH activity in a dose-dependent manner, activation in low and suppression in high dosage.

Above data suggest that ATP may contribute to PDH hyper-phosphorylation in cells. To test the possibility, the loss-of-function strategy was employed by pre-treatment of cells with the β-oxidation inhibitor, etomoxir (ET), which blocks acetyl-CoA production from palmitate. β-oxidation is required for ATP production from FFA. ATP was reduced by the ET pre-treatment (Fig 2D). The treatment abolished palmitate induction of PDH phosphorylation (Fig 2E). The data suggest that ATP elevation is required for the induction of PDH phosphorylation in cells.

### Direct induction of PDH hyper-phosphorylation by ATP

**Fig 3:**
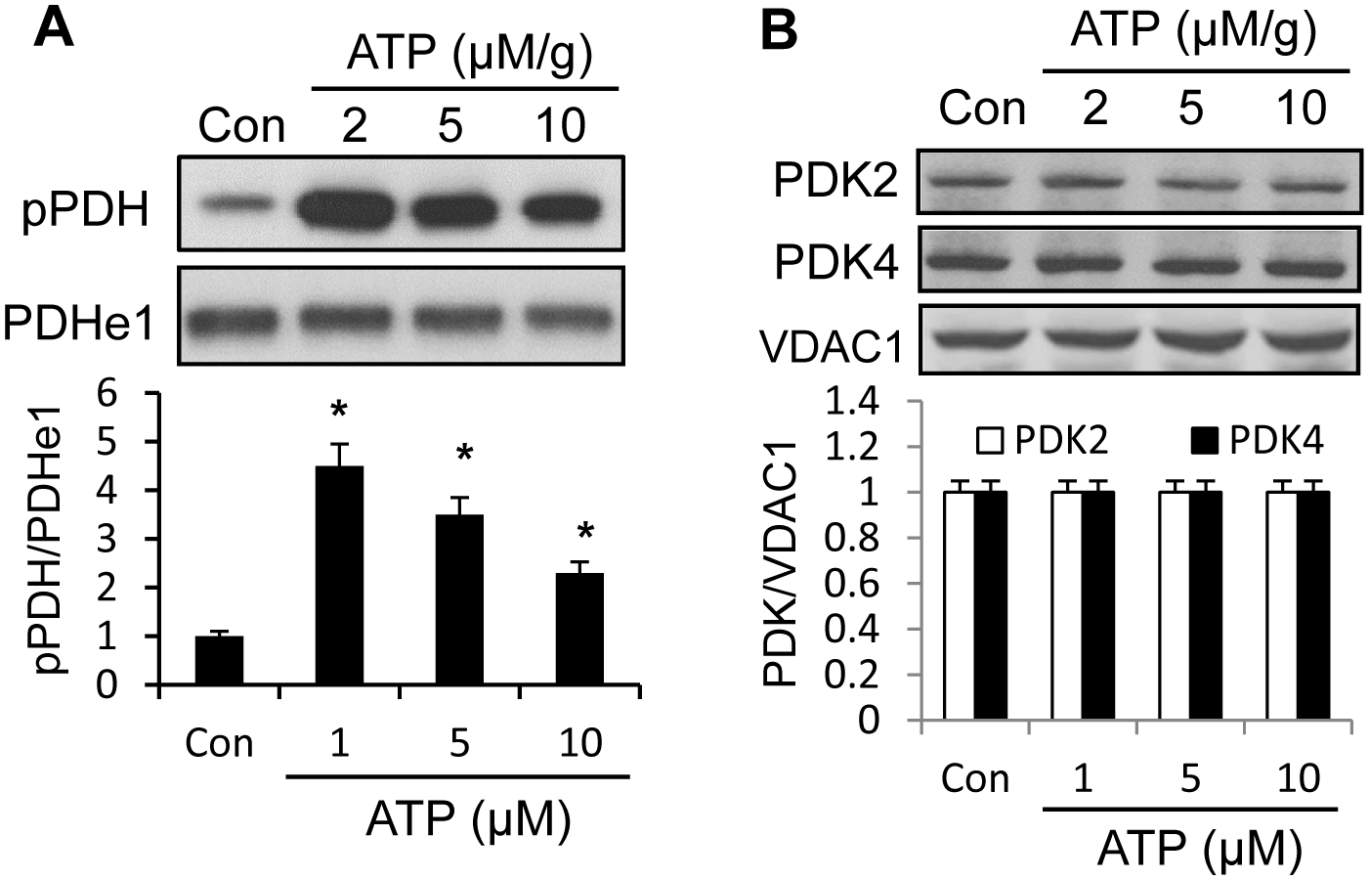
Induction of PDH phosphorylation by ATP. A. PDH phosphorylation. Mitochondria lysate was incubated with ATP at multiple dosages for 30 minutes at 37°C. PDH phosphorylation was quantified in Western blotting and the results are expressed in bar figure B. Protein levels of PDKs. The proteins of PDK2 and PDK4 were determined in Western blot and the signals were quantified. The experiments were performed three times with consistent results, and the representative blot is shown. In the bar figure, the result represents mean ± SE (n = 3). * p<0.05 compared with the control.

To test ATP in the induction of PDH phosphorylation, we treated mitochondrial lysate with ATP in vitro. Fresh mitochondria were isolated from cells and used in the preparation of mitochondria lysate. The internal ATP was depleted in the lysate by keeping the lysate at -70°C overnight. The lysate was then incubated with ATP at multiple concentrations at 37°C for 30 minutes to induce the phosphorylation. ATP was used at physiology relevant concentrations between 2–10 μM/g. PDH phosphorylation was induced by ATP at 2 μM/g and the ATP effect was reduced at the higher concentration (Fig 3A). The mechanism of less activity of ATP at higher concentrations is unknown. Activation of dephosphorylation reaction may play a role. Hepatocytes have two isoforms of PDKs, PDK2 and PDK4 [26]. The protein levels of PDK2 and PDK4 were examined and they were not altered by ATP (Fig 3B). The data suggests that ATP may induce PDH phosphorylation without altering PDK expression. The phosphorylation is unlikely a consequence of non-specific reaction. PDK is the only kinase to phosphorylate PDH.

### Induction of protein acetylation by ATP

**Fig 4:**
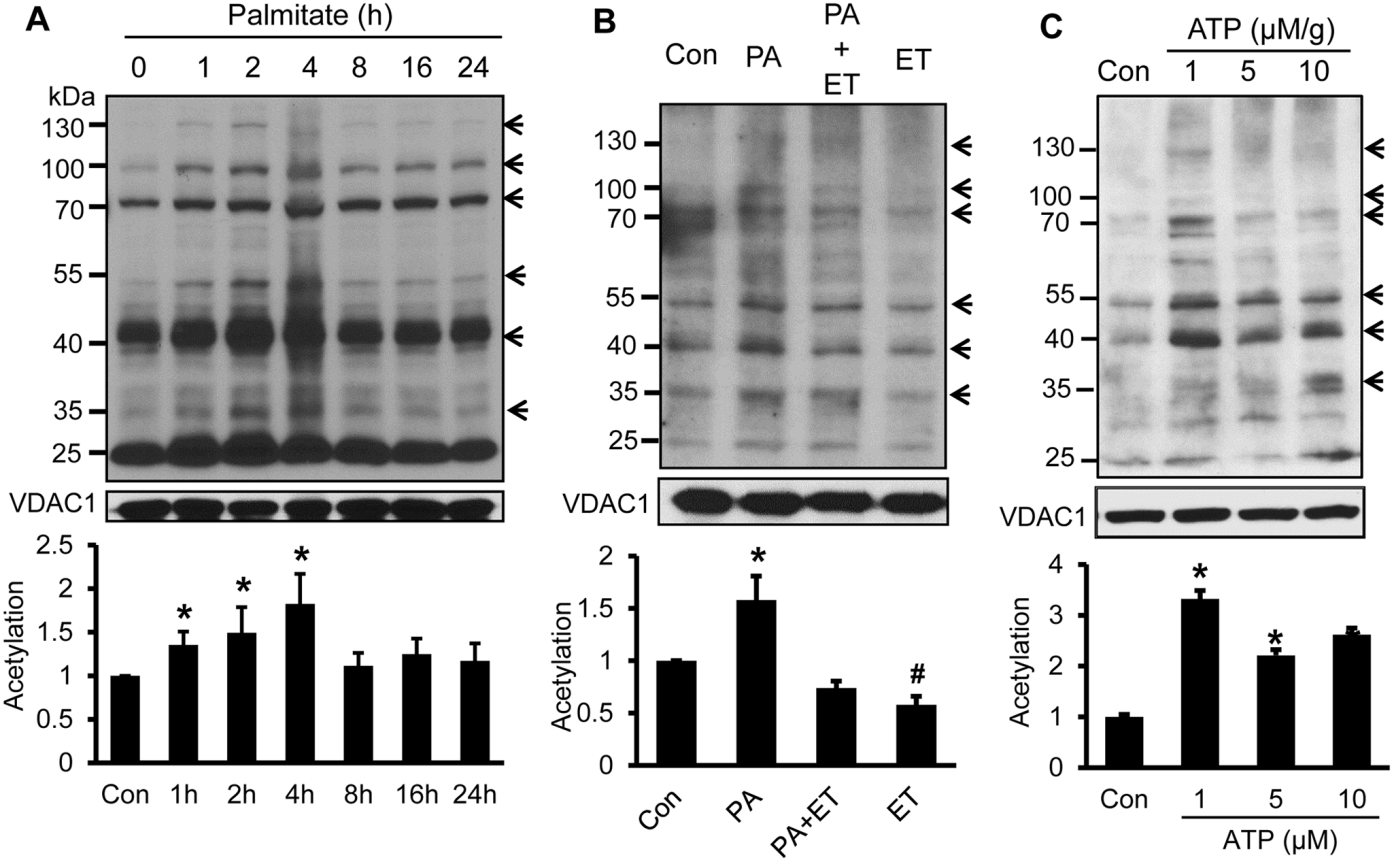
Induction of protein acetylation by ATP. A. Acetylation of mitochondrial protein. Cells were treated with palmitate (300 μM) in cell culture and acetylation was tested in isolated mitochondria at multiple time points. B. Acetylation inhibition by the β-oxidation inhibitor ET. The acetylation was determined in mitochondrial lysate following pre-treatment of cells with ET (50 μM) and palmitate (300 μM for 4 hr). C. Induction of acetylation by ATP. Mitochondrial lysate was incubated with ATP at multiple dosages for 30 minutes at 37°C. Acetylation was determined in the mitochondrial proteins by Western blotting. In this figure, the acetylation signal of highlighted bands was quantified individually, combined together and expressed in fold change in the bar figure. In the bar figure, the result represents mean ± SE (n = 3). * p<0.05 compared with the control (0 point).

Acetylation of mitochondrial proteins was examined in the mitochondrial lysate using the anti-acetyl-lysine antibody in Western blot. The acetylation was induced in 1c1c7 cells by palmitate treatment between 1–24 h. The induction was observed at multiple proteins as indicated by the band patterns (Fig 4A). The changed bands were highlighted by arrows on the right side of the blot. The molecular marker was on the left side. The signal in each band was quantified and accumulative signal strength in each lane was expressed in the bar figure under the blot. The acetylation was blocked by the β-oxidation inhibitor ET (Fig 4B), suggesting that the acetylation is dependent on ATP production. To test the possibility, ATP was used to treat the mitochondrial lysate through incubation in vitro (Fig 4C). An induction was observed in the protein acetylation following ATP treatment. The strongest acetylation was observed at the concentration of 1 μM/g ATP. The data suggest that ATP induces acetylation in mitochondria proteins.

### Acetyl-CoA exhibits a similar activity to ATP

**Fig 5:**
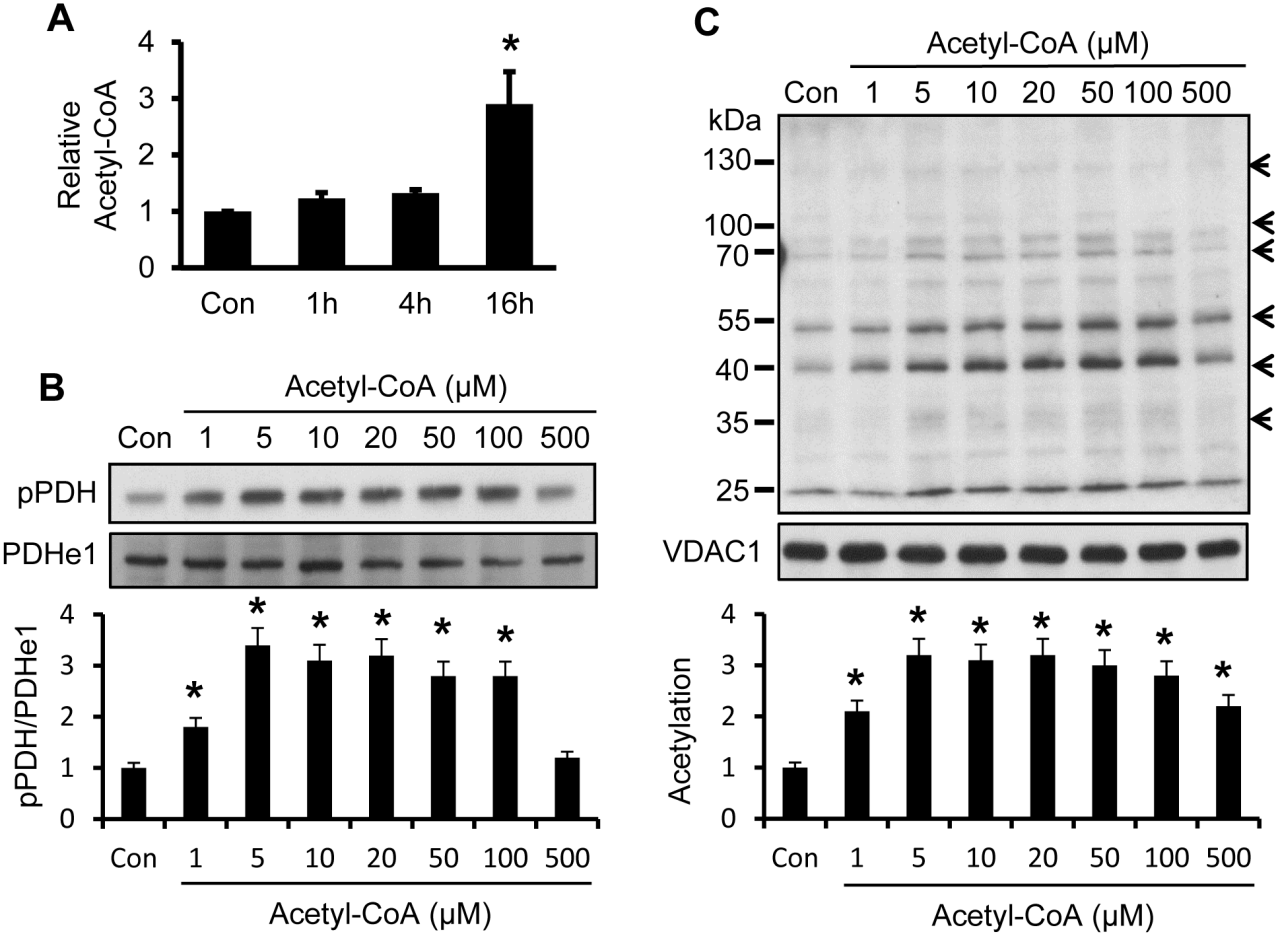
Induction of phosphorylation and acetylation by acetyl-CoA. A. Induction of acetyl-CoA in mitochondria by palmitate. Mitochondria were isolated from cells after palmitate treatment (300 μM) at 1, 4 and 16 h, and then used in the concentration assay of acetyl-CoA. B. Induction of PDH phosphorylation in mitochondria lysate by acetyl-CoA at different concentrations indicated. The phosphorylation was determined by Western blotting and the quantified signal was expressed in bar figure C. Induction of acetylation by acetyl-CoA. Mitochondria lysate was treated with acetyl-CoA at multiple dosages for 30 mins at 37°C. Acetylation was determined in mitochondrial protein by Western blotting. The experiments were performed three times with consistent results, and the representative blots are shown. The acetylation signal of highlighted bands was quantified individually, combined together and expressed in fold change in the bar figure. The result represents mean ± SE (n = 3). * p<0.05 compared with the control.

Above data suggest that the FFA effects are dependent on β-oxidation which breaks FFA down to generate acetyl-CoA. If this is true, acetyl-CoA should exhibit a similar activity to ATP in the induction of phosphorylation and acetylation. To test the possibility, acetyl-CoA was determined in the whole cell lysate after cell treatment by palmitate. An increase was observed at 1 h and the strong increase was observed at 16 h with the palmitate treatment (Fig 5A). Acetyl-CoA was used to treat mitochondria in a test tube to induce the protein modification. PDH phosphorylation was observed, and the strongest activity was observed at 5 μM of acetyl-CoA (Fig 5B). Acetylation of mitochondrial proteins was also increased by the treatment (Fig 5C). These data suggest that acetyl-CoA may mediate the palmitate activity in the induction of phosphorylation and acetylation.

### Protection of mitochondria function with the β-oxidation inhibitor

**Fig 6:**
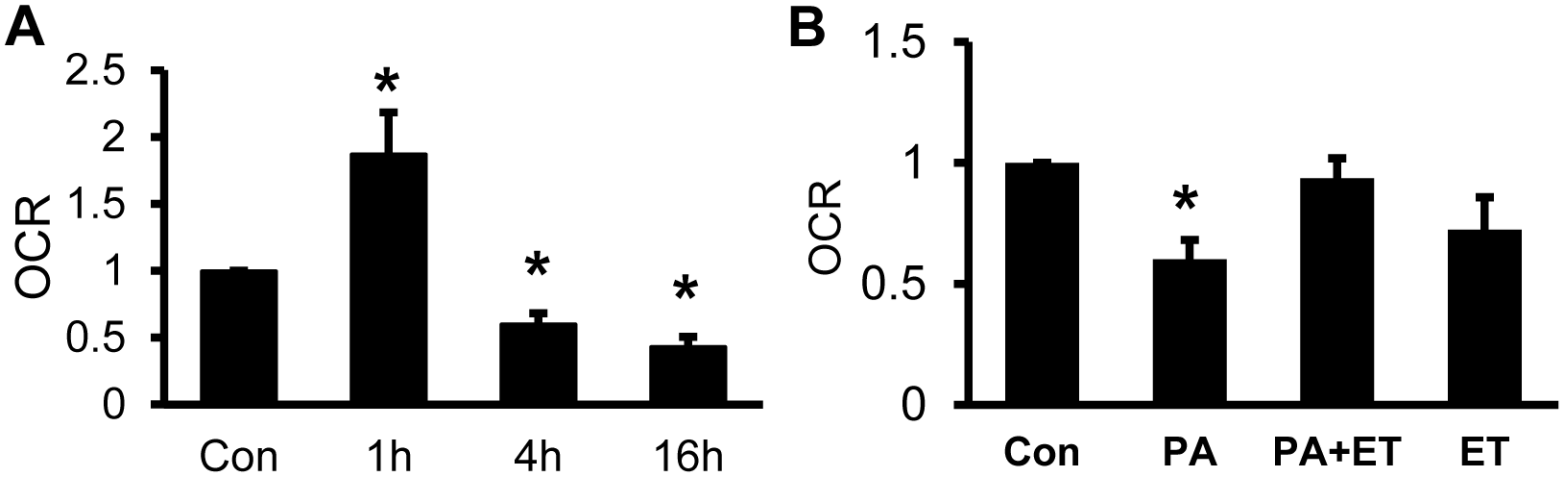
Protection of mitochondria function by β-oxidation inhibitor. A. Inhibition of oxygen consumption rate (OCR) by palmitate. OCR was examined in mitochondria after palmitate treatment of cells at times indicated. B. Protection of mitochondrial function by Etomoxir (ET, 50 μM). ET was used to block β-oxidation in cells in the presence of palmitate (300 μM, 4 h). Freshly isolated mitochondria were used in the assays. In the bar figure, the result represents mean ± SE (n = 3). * p<0.05 compared with the control.

The phosphorylation and acetylation may contribute to mitochondrial inhibition by palmitate in our system. To test this possibility, oxygen consumption rate (OCR) was examined using the Seahorse system to determine the mitochondrial function. Mitochondria were isolated from cells immediately after palmitate treatment and tested for OCR in the presence of substrate of succinate. OCR was induced at 1 h, but suppressed at 4 h and 16 h by palmitate (Fig 6A). The palmitate activity was blocked by inhibition of β-oxidation with ET (Fig 6B). The data suggests that the inhibition may be a result of palmitate induction of the hyper-phosphorylation and hyper-acetylation.

## Discussion

Our data suggest that ATP induces PDH phosphorylation independently of PDK expression. This mechanism may be used by FFA to inhibit glucose utilization in mitochondria given that FFA has a high density in energy. The inhibition is mediated by PDH hyper-phosphorylation as suggested by our data. PDH is subject to reversible phosphorylation in physiological conditions upon changes in fuel supply. The phosphorylation is catalyzed by PDK. Hyper-phosphorylation of PDH is a mechanism of enzyme inhibition in the liver and muscle in starvation and diabetes [5–8], which may contribute to the reduction in glucose utilization by those organs. Treatment of cells with palmitate mimics the starvation condition in the high level of FFA [8, 27]. Palmitate is the most abundant FFA in the blood. An increase in PDK expression is a common mechanism for elevation in PDH phosphorylation in those conditions [28, 29]. However, the expression independent mechanism was not known. Our data suggest that the phosphorylation may happen independently of PDK expression. The mechanism is elevation of intracellular ATP. This possibility is supported by the increased ATP in the liver of DIO mice and in cells treated with palmitate. In vitro, PDH phosphorylation was induced by ATP in the incubation with the mitochondrial lysate, which was observed in the absence of PDK2 and PDK4 protein elevation. As a substrate, ATP likely activates PDK to induce the phosphorylation. The ATP effect was observed at 2 μM/g protein, which is in the physiological range of ATP concentrations (1–3 μM/g) in mouse liver [30]. The ATP activity in cells is supported by the etomoxir effect on inhibition of palmitate-induced PDH phosphorylation. This group of data suggests that ATP may directly induce PDH phosphorylation in vivo to limit glucose utilization by mitochondria.

In addition to phosphorylation, ATP promoted protein acetylation in mitochondria in our system. Hyperacetylation represents a mechanism of inhibition of glucose utilization by mitochondria in SIRT3 knockout mice [15–17]. In current study, we observed that the acetylation was induced by ATP in liver, cells and mitochondria lysate. Interestingly, the acetylation patterns were highly consistent in the three conditions. Inhibition of ATP production by etomoxir prevented the acetylation. Inhibition of β-oxidation is a mechanism of etomoxir activity in the improvement of glucose metabolism in vivo [31, 32]. It was unknown if the acetylation involves in the etomoxir activity. Our data suggest that the inhibition of mitochondrial protein acetylation may represent a new mechanism of etomoxir activity in the regulation of glucose metabolism. In addition to ATP, acetyl-CoA also induced the acetylation. Our data suggest that as substrates, ATP and acetyl-CoA may activate acetyl-transferase in the acetylation reaction although the nature of transferase remains unknown. This study suggests that ATP may inhibit glucose utilization by mitochondria through induction of the acetylation.

In summary, we investigated molecular mechanism by which ATP inhibits mitochondrial activity with a focus on posttranslational modification of proteins by phosphorylation and acetylation. The role of ATP is supported by several lines of evidence including those from DIO mice, palmitate-treated cells, β-oxidation inhibition by etomoxir, and ATP treatment of mitochondrial lysate. The data consistently suggest that ATP is able to induce the phosphorylation and acetylation. Although the nature of acetylated proteins and acetyltransferase remains unknown in this study, this does not influence our conclusion about ATP in the inhibition of mitochondrial function. It is well known that AMPK is down-regulated by ATP surplus in the feedback regulation of mitochondria function. Current study does not exclude the role of the AMPK pathway. However, we propose that as an AMPK-independent pathway, the PDH response may contribute to the feedback regulation as well. In addition, the acetylation of mitochondrial proteins may represent another AMPK-independent feedback mechanism. The study provides a mechanism for insulin resistance in the condition of ATP surplus in human and mouse [10, 11]. Our data suggest a new mechanism for the insulin-sensitizing activities of anti-diabetic medicines such as metformin and berberine, which inhibit ATP production in mitochondria [20].

## References

[1] Randle PJ, Garland PB, Hales CN, Newsholme EA. The glucose fatty-acid cycle. Its role in insulin sensitivity and the metabolic disturbances of diabetes mellitus. Lancet. 1963;1(7285):785–9. Epub 1963/04/13. . 13990765 10.1016/s0140-6736(63)91500-9

[2] Ruderman NB, Carling D, Prentki M, Cacicedo JM. AMPK, insulin resistance, and the metabolic syndrome. J Clin Invest. 2013;123(7):2764–72. 10.1172/JCI67227 23863634 PMC3696539

[3] Hardie DG, Ross FA, Hawley SA. AMPK: a nutrient and energy sensor that maintains energy homeostasis. Nat Rev Mol Cell Biol. 2012;13(4):251–62. Epub 2012/03/23. 10.1038/nrm3311 . 22436748 PMC5726489

[4] Kerbey AL, Radcliffe PM, Randle PJ, Sugden PH. Regulation of kinase reactions in pig heart pyruvate dehydrogenase complex. Biochem J. 1979;181(2):427–33. 227365 10.1042/bj1810427 PMC1161174

[5] Patel MS, Korotchkina LG. Regulation of the pyruvate dehydrogenase complex. Biochem Soc Trans. 2006;34(Pt 2):217–22. 10.1042/BST20060217 . 16545080

[6] Jeoung NH, Wu P, Joshi MA, Jaskiewicz J, Bock CB, Depaoli-Roach AA, et al Role of pyruvate dehydrogenase kinase isoenzyme 4 (PDHK4) in glucose homoeostasis during starvation. Biochem J. 2006;397(3):417–25. Epub 2006/04/12. BJ20060125 [pii] 10.1042/BJ20060125 16606348 PMC1533314

[7] Pilegaard H, Birk JB, Sacchetti M, Mourtzakis M, Hardie DG, Stewart G, et al PDH-E1α Dephosphorylation and Activation in Human Skeletal Muscle During Exercise. Diabetes. 2006;55(11):3020–7. 10.2337/db06-0152 17065338

[8] Wu P, Inskeep K, Bowker-Kinley MM, Popov KM, Harris RA. Mechanism responsible for inactivation of skeletal muscle pyruvate dehydrogenase complex in starvation and diabetes. Diabetes. 1999;48(8):1593–9. . 10426378 10.2337/diabetes.48.8.1593

[9] Jing E, O'Neill BT, Rardin MJ, Kleinridders A, Ilkeyeva OR, Ussar S, et al Sirt3 regulates metabolic flexibility of skeletal muscle through reversible enzymatic deacetylation. Diabetes. 2013;62(10):3404–17. 10.2337/db12-1650 23835326 PMC3781465

[10] Nair KS, Bigelow ML, Asmann YW, Chow LS, Coenen-Schimke JM, Klaus KA, et al Asian Indians have enhanced skeletal muscle mitochondrial capacity to produce ATP in association with severe insulin resistance. Diabetes. 2008;57(5):1166–75. Epub 2008/02/21. db07-1556 [pii] 10.2337/db07-1556 . 18285554 PMC7812549

[11] Choi CS, Befroy DE, Codella R, Kim S, Reznick RM, Hwang YJ, et al Paradoxical effects of increased expression of PGC-1alpha on muscle mitochondrial function and insulin-stimulated muscle glucose metabolism. Proc Natl Acad Sci U S A. 2008;105(50):19926–31. Epub 2008/12/11. 0810339105 [pii] 10.1073/pnas.0810339105 19066218 PMC2598730

[12] Lin Y-y, Kiihl S, Suhail Y, Liu S-Y, Chou Y-h, Kuang Z, et al Functional dissection of lysine deacetylases reveals that HDAC1 and p300 regulate AMPK. Nature. 2012;482(7384):251–5. http://www.nature.com/nature/journal/v482/n7384/abs/nature10804.html#supplementary-information. 10.1038/nature10804 22318606 PMC3277212

[13] Zhao S, Xu W, Jiang W, Yu W, Lin Y, Zhang T, et al Regulation of cellular metabolism by protein lysine acetylation. Science. 2010;327(5968):1000–4. Epub 2010/02/20. 327/5968/1000 [pii] 10.1126/science.1179689 . 20167786 PMC3232675

[14] Wang Q, Zhang Y, Yang C, Xiong H, Lin Y, Yao J, et al Acetylation of metabolic enzymes coordinates carbon source utilization and metabolic flux. Science. 2010;327(5968):1004–7. Epub 2010/02/20. 327/5968/1004 [pii] 10.1126/science.1179687 . 20167787 PMC4183141

[15] Hirschey MD, Shimazu T, Goetzman E, Jing E, Schwer B, Lombard DB, et al SIRT3 regulates mitochondrial fatty-acid oxidation by reversible enzyme deacetylation. Nature. 2010;464(7285):121–5. http://www.nature.com/nature/journal/v464/n7285/suppinfo/nature08778_S1.html. 10.1038/nature08778 20203611 PMC2841477

[16] Shimazu T, Hirschey MD, Hua L, Dittenhafer-Reed KE, Schwer B, Lombard DB, et al SIRT3 Deacetylates Mitochondrial 3-Hydroxy-3-Methylglutaryl CoA Synthase 2 and Regulates Ketone Body Production. Cell Metabolism. 2010;12(6):654–61. 10.1016/j.cmet.2010.11.003 21109197 PMC3310379

[17] Hirschey MD, Shimazu T, Jing E, Grueter CA, Collins AM, Aouizerat B, et al SIRT3 deficiency and mitochondrial protein hyperacetylation accelerate the development of the metabolic syndrome. Mol Cell. 2011;44(2):177–90. 10.1016/j.molcel.2011.07.019 21856199 PMC3563434

[18] Perry RJ, Camporez JP, Kursawe R, Titchenell PM, Zhang D, Perry CJ, et al Hepatic acetyl CoA links adipose tissue inflammation to hepatic insulin resistance and type 2 diabetes. Cell. 2015;160(4):745–58. 10.1016/j.cell.2015.01.012 . 25662011 PMC4498261

[19] Yin J, Gao Z, Liu D, Liu Z, Ye J. Berberine Improves Glucose Metabolism through Induction of Glycolysis. Am J Physiol Endocrinol Metab. 2008;294:E148–E56. . 17971514 10.1152/ajpendo.00211.2007 PMC2464622

[20] Zhang Y, Ye J. Mitochondrial inhibitor as a new class of insulin sensitizer. Acta Pharmaceutica Sinica B 2012;4:341–9. 10.1016/j.apsb.2012.06.010 PMC3660979 23710432

[21] Hawley SA, Ross FA, Chevtzoff C, Green KA, Evans A, Fogarty S, et al Use of cells expressing gamma subunit variants to identify diverse mechanisms of AMPK activation. Cell Metab. 2010;11(6):554–65. Epub 2010/06/04. S1550-4131(10)00112-9 [pii] 10.1016/j.cmet.2010.04.001 . 20519126 PMC2935965

[22] Tang T, Zhang J, Yin J, Staszkiewicz J, Gawronska-Kozak B, Mynatt R, et al Uncoupling of Inflammation and Insulin Resistance by NF-kB in Transgenic Mice through Induction of Energy Expenditure. J Biol Chem. 2010;285:4637–44. Epub Online Dec 17th 2009. 10.1074/jbc.M109.068007 20018865 PMC2836069

[23] Xu F, Gao Z, Zhang J, Rivera CA, Yin J, Weng J, et al Lack of SIRT1 (Mammalian Sirtuin 1) Activity Leads to Liver Steatosis in the SIRT1+/− Mice: A Role of Lipid Mobilization and Inflammation. Endocrinology. 2010;151 (6):2504–14. Epub March 25, 2010. 10.1210/en.2009-1013 20339025 PMC2875813

[24] Frezza C, Cipolat S, Scorrano L. Organelle isolation: functional mitochondria from mouse liver, muscle and cultured fibroblasts. Nat Protoc. 2007;2(2):287–95. Epub 2007/04/05. 10.1038/nprot.2006.478 . 17406588

[25] Gao Z, He Q, Peng B, Chiao PJ, Ye J. Regulation of Nuclear Translocation of HDAC3 by IkBa Is Required for Tumor Necrosis Factor Inhibition of Peroxisome Proliferator-activated Receptor {gamma} Function. J Biol Chem. 2006;281(7):4540–7. . 16371367 10.1074/jbc.M507784200 PMC1447600

[26] Holness MJ, Bulmer K, Smith ND, Sugden MC. Investigation of potential mechanisms regulating protein expression of hepatic pyruvate dehydrogenase kinase isoforms 2 and 4 by fatty acids and thyroid hormone. Biochem J. 2003;369(Pt 3):687–95. 10.1042/BJ20021509 12435272 PMC1223128

[27] Huang B, Wu P, Bowker-Kinley MM, Harris RA. Regulation of pyruvate dehydrogenase kinase expression by peroxisome proliferator-activated receptor-alpha ligands, glucocorticoids, and insulin. Diabetes. 2002;51(2):276–83. . 11812733 10.2337/diabetes.51.2.276

[28] Roche TE, Hiromasa Y. Pyruvate dehydrogenase kinase regulatory mechanisms and inhibition in treating diabetes, heart ischemia, and cancer. Cell Mol Life Sci. 2007;64(7–8):830–49. 10.1007/s00018-007-6380-z . 17310282 PMC11136253

[29] Holness MJ, Bulmer K, Gibbons GF, Sugden MC. Up-regulation of pyruvate dehydrogenase kinase isoform 4 (PDK4) protein expression in oxidative skeletal muscle does not require the obligatory participation of peroxisome-proliferator-activated receptor alpha (PPARalpha). Biochem J. 2002;366(Pt 3):839–46. 10.1042/BJ20020754 12099888 PMC1222844

[30] Berglund ED, Lee-Young RS, Lustig DG, Lynes SE, Donahue EP, Camacho RC, et al Hepatic energy state is regulated by glucagon receptor signaling in mice. J Clin Invest. 2009;119(8):2412–22. 19662685 10.1172/JCI38650 PMC2719934

[31] Oakes ND, Cooney GJ, Camilleri S, Chisholm DJ, Kraegen EW. Mechanisms of liver and muscle insulin resistance induced by chronic high-fat feeding. Diabetes. 1997;46(11):1768–74. Epub 1997/11/14. . 9356024 10.2337/diab.46.11.1768

[32] Horn CC, Ji H, Friedman MI. Etomoxir, a fatty acid oxidation inhibitor, increases food intake and reduces hepatic energy status in rats. Physiology & behavior. 2004;81(1):157–62. Epub 2004/04/03. 10.1016/j.physbeh.2004.01.007 . 15059695

